# Leveraging Learning Analytics to Model Student Engagement in Graduate Statistics: A Problem-Based Learning Approach in Agricultural Education †

**DOI:** 10.3390/bs15101360

**Published:** 2025-10-05

**Authors:** Zhihong Xu, Fahmida Husain Choudhury, Shuai Ma, Theresa Pesl Murphrey, Kim E. Dooley

**Affiliations:** Department of Agricultural Leadership, Education and Communications, Texas A&M University, College Station, TX 77843, USA; fahmida15.alec@tamu.edu (F.H.C.); shuai_ma2021@tamu.edu (S.M.); t-murphrey@tamu.edu (T.P.M.); k-dooley@tamu.edu (K.E.D.)

**Keywords:** learning management system (LMS), problem-based learning (PBL), student engagement, self-efficacy, diverse learners, mixed methods

## Abstract

Graduate students often experience difficulties in learning statistics, particularly those who have limited mathematical backgrounds. In recent years, Learning Management Systems (LMS) and Problem-Based Learning (PBL) have been widely adopted to support instruction, yet little research has explored how these tools relate to learning outcomes using mixed methods design. Limited studies have employed machine learning methods such as clustering analysis in Learning Analytics (LA) to explore different behavior of clusters based on students log data. This study followed an explanatory sequential mixed methods design to examine student engagement patterns on Canvas and learning outcomes of students in a graduate-level statistics course. LMS log data and surveys were collected from 31 students, followed by interviews with 19 participants. K-means clustering revealed two groups: a high-performing group with lower LMS engagement and a low-performing group with higher LMS engagement. Six themes emerged from a thematic analysis of interview transcripts: behavioral differences in engagement, the role of assessment, emotional struggle, self-efficacy, knowledge or skill gain, and structured instructional support. Results indicated that low-performing students engaged more frequently and benefited from structured guidance and repeated exposure. High-performing students showed more proactive and consistent engagement habits. These findings highlight the importance of intentional course design that combines PBL with LMS features to support diverse learners.

## 1. Introduction and Literature Review

Statistical literacy is essential for graduate students, though it is the course in which most of the students face challenges and difficulties. It is an old scientific discipline, but its application has never been more topical (Zorić, 2021). Scientific knowledge advancement and policy decision-making rely heavily on data analysis across various fields, including the social sciences and agricultural sciences, where statistics plays a crucial role (Fienberg, 2014). It enables researchers and professionals to identify patterns, test hypotheses, enhance efficiency, and make data-driven improvements in various fields (Makkawi & Abdelrahman, 2016). Despite its importance, many graduate students, especially those from non-statistical backgrounds—experience anxiety, struggle with the material, and underestimate its relevance (DeVaney, 2010). Negative perceptions and fear of failure often hinder learning and skill development.

A learning management system (LMS) is a digital platform that supports teaching and learning by providing tools for delivering content, tracking progress, and facilitating communication between instructors and students (Hariri, 2014). Engagement of students plays a significant role in learning outcomes, yet the possible ways through which students interact with the Learning Management System (LMS) remain underexplored, particularly in the context of Problem-Based Learning (PBL). PBL is a student-centered instructional approach in which learners acquire knowledge and skills by collaboratively solving real-world problems rather than passively receiving information (Barrows, 1986). Hariri (2014) found that LMS facilitates online learning, improves communication between instructors and students, and provides various features to support the learning process. Students generally have positive perceptions of LMS, recognizing their benefits in facilitating learning and understanding course content (Hariri, 2014). Research about online learning platforms has shown that students’ satisfaction is shaped by their computer self-efficacy and the perceived ease of use and usefulness of these systems, underscoring the importance of user-friendly design for supporting engagement (Jiang et al., 2021). LMSs are becoming increasingly important in supporting 21st-century learning, with recommendations for user-friendly interfaces and larger storage capacities to facilitate active and collaborative learning (Rahman et al., 2019).

The field of Learning Analytics (LA), including LMS log data, is closely related to LMS use, which involves collecting and analyzing student data within digital learning environments (Siemens, 2012). It can support personalized learning, improve instructional effectiveness, and help identify at-risk learners (Banihashem et al., 2018). Research on LMS and student performance presented mixed results, with some studies highlighting benefits while others emphasized the influence of additional factors. According to Broadbent (2016), combining LA and LMS can positively impact academic success, depending on various factors beyond the mere frequency of use. He argued that psychological factors, such as self-efficacy, are more important for academic success than mere LMS participation. However, Avcı and Ergün (2019) contradicted this claim, showing that student engagement and academic performance improved through high LMS participation, a finding further supported by Ojo (2024).

To better understand student engagement, researchers have applied advanced analytics techniques such as clustering and sequence analysis. These methods help categorize students into low-, moderate-, and high-engagement groups, each with distinct behavior profiles and academic outcomes (Lu et al., 2017; Larrabee Sønderlund et al., 2019). Key variables for identifying engagement patterns include login frequency, total time on the platform, number of clicks, quiz attempts, and forum activity. Researchers typically use performance metrics, such as course grades or assessment scores, to evaluate these clusters.

Student engagement is widely understood as a multidimensional construct that includes behavioral, emotional, and cognitive components, each contributing to learning processes in different ways (Fredricks et al., 2004). Behavioral engagement involves participation and sustained effort, emotional engagement reflects interest, enjoyment, or a sense of belonging, and cognitive engagement refers to investment in learning and the use of deep learning strategies. Recognizing these dimensions is important for understanding how students interact with LMS-supported environments and respond to instructional approaches (Kahu et al., 2020).

Beyond these behavior metrics, the concept of engagement includes how students feel and think about their learning (Kahu et al., 2020). Students exhibit effort regulation by staying focused and continuing to work even when it is hard or when there are distractions (MacIntyre et al., 2021). Both are important for learning, especially in online environments. Many studies have reported the use LMS log data to measure engagement. For example, researchers have used metrics such as time spent reading materials or watching videos, the number of clicks, quiz attempts, and login frequency. One study showed that students who read for a longer time had better performance (Yamada et al., 2017). Other research found that students who missed activities or rarely interacted with the LMS had lower grades, while students who used it more regularly performed better (Bulut et al., 2022; Ramirez-Arellano et al., 2019). Further, researchers reported that students’ confidence and study habits improved when they were provided feedback from dashboards (Karaoglan Yilmaz, 2022).

High-performing students tend to demonstrate regular LMS usage and active participation in forums, whereas low-performing students often display inconsistent engagement or procrastination (Gašević et al., 2015). Interestingly, some studies suggest that students with lower digital literacy or confidence may benefit more from LMS-supported instruction, potentially due to increased exposure and structure (Le et al., 2022). These findings highlight the need to consider individual differences in digital readiness and learning preferences.

Despite the usefulness of behavior data, learning analytics alone cannot fully explain student success. While LMS log data reveals what students do, they do not capture why students behave that way or how students perceive their learning experience. Studies such as Redmond et al. (2023) and Kahu et al. (2020) have shown that emotional support, course structure, and personal circumstances significantly affect students’ engagement and outcomes. These findings suggest that understanding students’ perceptions—particularly how they respond to instructional strategies—requires more than log data.

LMS platforms offer structured spaces for communication, collaboration, and resource sharing that can support more interactive forms of learning (Hariri, 2014). Learning analytics from LMS log data allows instructors to monitor engagement and progress, offering insights into how students respond to different tasks (Rienties et al., 2015). These features make LMS- and LA-supported environments well suited for implementing student-centered approaches such as problem-based learning, where instructors can use analytics to guide facilitation and provide timely feedback to support students’ learning.

To address this gap, pedagogical frameworks such as problem-based learning (PBL) offer valuable insights. PBL is a student-centered pedagogy where students acquire knowledge by actively engaging with real-world problems instead of passively receiving information (Barrows, 1986). It was initially developed for medical education but has since been widely applied to various disciplines, including engineering, business, and social sciences, where the role of the instructor shifts from a content provider to a facilitator, offering support, guidance, and feedback while monitoring students’ learning progress (Schmidt et al., 2011).

In graduate statistics courses within agricultural education, PBL fosters student engagement through group collaboration, reflection, and analytical reasoning. It also aligns with students’ needs to develop critical thinking and real-world problem-solving skills. An important dimension of evaluating PBL effectiveness is students perceived learning outcomes—how students evaluate their own learning gains, including their understanding of course content and the development of critical skills (Rust, 2002). One of the most studied aspects of perceived learning is self-efficacy, which Bandura (1997) defined as individuals’ beliefs in their capacity to perform specific academic tasks. Self-efficacy influences motivation, effort, and persistence—especially in statistical learning, where students often feel overwhelmed or anxious.

Prior research has shown that students exposed to PBL often achieve better outcomes compared to traditional instruction. For example, Bihari et al. (2021) found that students using PBL in biostatistics scored higher across assessments. Similarly, Bukumiric et al. (2022) and Kurniawan and Perdana (2019) reported improved statistical reasoning and performance under PBL methods. Learning analytics can provide insights into how students interact with problem-based tasks and whether these engagement patterns are linked to academic success (Rienties et al., 2015).

While outcomes can be measured through grades, students’ perceptions provide important context. Some research shows that students with higher engagement levels often report more confidence and deeper learning (e.g., Redmond et al., 2023). In contrast, lower-performing or less engaged students may perceive learning differently, even in the same environment. These differences in perception across student groups highlight the value of including both quantitative and qualitative insights when evaluating PBL’s impact.

Many studies have used LMS log data to examine student engagement, often focusing on behavior indicators such as clicks, time spent, or login frequency (Lu et al., 2017; Larrabee Sønderlund et al., 2019; Bulut et al., 2022). While these measures capture what students do, they do not explain why students behave that way or how they feel about their learning (Redmond et al., 2023; Kahu et al., 2020). Very few studies have combined learning analytics with qualitative interviews to link behavior with perception, and even fewer have examined how students at different performance levels experience PBL within LMS-supported environments.

To address this gap, the present study employed a mixed-methods design in which quantitative data reveal engagement patterns and outcomes, while qualitative interviews provide insight into students’ perceptions and the mechanism shaping those patterns (Creswell & Plano Clark, 2018). Research on emerging technologies such as virtual and augmented reality in STEM education further underscores the value of technology-enhanced, student-centered approaches, offering directions for future investigation (Jiang et al., 2025).

By investigating LMS data with student interviews, this study compares the behaviors and perceptions of low- and high-performing students in a PBL-based statistics course. The research offers a more comprehensive understanding of how learners engage with PBL-supported statistics instruction by using this approach. Including student voices helps explain the meaning behind engagement patterns and offers guidance for improving instruction to support diverse learners.

### 1.1. Purpose and Objectives

Understanding how students engage in graduate statistics education is crucial for enhancing learning outcomes. The integration of PBL and LMS provides a framework for examining student interactions, instructional support, and academic performance. Educators can identify distinct engagement patterns among students with different performance levels by using learning analytics, which can inform more targeted and effective instructional strategies.

This study adopted a mixed-methods design, combining LMS log data with qualitative interviews to explore both engagement behavior and students’ perceptions of learning. By integrating these two strands of evidence, the study aimed to offer a more comprehensive understanding of how students with varying levels of academic performance experience and interpret PBL- and LMS- supported instruction.

### 1.2. Research Questions

RQ1: What are distinct clusters or groups of students that emerge based on LMS interaction patterns and academic performance?
RQ2: How do engagement behaviors and perceptions of effort regulation differ between these groups?
RQ3: How do students from different performance groups perceive their learning outcome—such as the impact of PBL and their self-efficacy—how do they interpret these effects?

## 2. Materials and Methods

This study employed an explanatory sequential mixed-methods design (Creswell & Plano Clark, 2018), in which quantitative data was collected and analyzed first, followed by qualitative interviews to interpret and expand on the initial findings. This design allowed for modeling student engagement behavior using learning analytics and for gaining deeper insights into students’ learning experiences through interviews. The approach provided researchers a comprehensive understanding of both observable behavioral patterns and underlying perceptions, particularly within a PBL environment supported by an LMS.

### 2.1. Participants

Participants were graduate students at Texas A&M University enrolled in a graduate-level statistics course within the agricultural sciences program. Recruitment occurred in class, where students were informed of the study’s purpose, its voluntary nature, confidentiality, and approval by the Texas A&M University Institutional Review Board (IRB). The intervention spanned three semesters—spring 2023 (*n* = 10), fall 2023 (*n* = 13), and fall 2024 (*n* = 8)—for a total of 31 students who consented to share their Canvas log data, survey data, and academic performance for the quantitative phase.

To be eligible for the interview, a participant had to be enrolled in the class and consent to participate in the semi-structured interview. The interviews were conducted at two time points: mid-and end of semester, yielding 37 interviews in total. Eighteen students completed both midterm and end-of-semester interviews, and one student completed only the end-of-semester interview. The researcher who conducted the interviews was a member of the research team rather than the course instructor. Each interview lasted approximately 30 min.

The sampling strategy was inclusive of all enrolled students, which reduced concerns about selection bias within the class context. Although 12 students declined to be interviewed, the 19 participants who completed the interview represented a range of performance levels, providing variation in perspectives.

### 2.2. PBL Instruction Design

The course followed a structured PBL model (Chung, 2019) in which students worked on real-world data problems that encouraged critical thinking, applied analysis, and collaborative learning. Instructional activities included lectures, live coding sessions using R (Version 4.3.0), group discussions, and project-based work. Canvas, the university’s LMS, was used to organize and deliver instructional materials, post announcements, facilitate feedback, and monitor student engagement. Materials such as lecture slides and recordings, R code files, handouts, and graded assignments were accessible via Canvas.

### 2.3. Measures

#### 2.3.1. Quantitative Measures

LMS log data were collected biweekly from all 31 graduate students to enable examination of students’ engagement behavior. Log entries were categorized into three interaction types based on Moore (1989) and Hillman et al. (1994): (1) Interaction with the system (e.g., clicks on the course homepage, changes to notification preferences, views on the Canvas mobile app, and use of the course calendar); (2) Interaction with content (e.g., accessing course materials such as the syllabus, class slides, R code files, and module pages); (3) Interaction with the instructor (e.g., submitting assignments and quizzes, posting to discussion boards, and viewing instructor announcements or feedback).

The completion of a pre-survey and post-survey allowed researchers to measure learning outcomes and perceptions of the PBL approach. The pre-survey included items on motivation (Pintrich & De Groot, 1990) and statistical self-efficacy (Pathirage, 2015). The post-survey retained the self-efficacy scale and added perceived impact of PBL (Lee, 2010), along with cognitive presence, social presence and teaching presence from the Community of Inquiry framework (Arbaugh et al., 2008). All items used a 5-point Likert scale (1 = “strongly disagree” or “not confident at all” to 5 = “strongly agree” or “strongly confident”). Prior studies have shown high internal consistency for these measures (Cronbach’s alpha = 0.68 to 0.97); and in this study, overall reliability was α = 0.92. The reliabilities were as follows: 0.65 for motivation scale, 0.97 for self-efficacy post, 0.80 for impact of PBL, 0.77 for cognitive presence, 0.81 for social presence, and 0.85 for teaching presence. Students’ course exam scores were used to assess academic performance.

#### 2.3.2. Qualitative Measures

Semi-structured interviews were conducted via Zoom, each lasting approximately 30 min. An interview protocol developed by the researchers guided the conversation, focusing on student engagement behaviors, emotional experiences, and perceptions of learning outcomes related to PBL and LMS use. Interviews were purposefully conducted at two time points: mid-semester and end-of-semester.

### 2.4. Data Analysis

#### 2.4.1. Quantitative Analysis

Descriptive statistics were utilized to summarize LMS interaction patterns. K-means clustering, a machine learning technique, was employed to group students based on engagement data (i.e., Canvas Log data) and academic performance. Independent sample t-tests were then conducted to examine group differences. All statistical analyses were conducted using RStudio (version 4.3.0; R Core Team, 2022).

#### 2.4.2. Qualitative Analysis

All interviews were audio-recorded with participant consent and transcribed verbatim. A total of 37 transcripts were analyzed using thematic analysis following Clarke and Braun’s (2006) six-phase process. The process included open coding to identify meaningful segments, followed by axial coding to categorize themes, and synthesis aligned with research questions. The analysis approach was guided by qualitative research principles outlined by Tisdell et al. (2025). After the manual coding process, MAXQDA 24 was used to organize coded segments and generate visualizations, such as code matrix browsers and profile comparison charts, which are included in Section 3 to illustrate thematic variation across student groups.

### 2.5. Trustworthiness and Reflexivity

To enhance the credibility and interpretive depth of the qualitative findings, the researchers engaged in reflexive memo writing and sought peer feedback throughout the process. Trustworthiness was established using Lincoln and Guba’s (1985) criteria: credibility, dependability, transferability, and confirmability. Researchers maintained an audit trail to document analytic decisions and ensure transparency.

## 3. Results and Discussions

### 3.1. Distinct Engagement Patterns and Performance Groups (RQ1)

**Figure 1:**
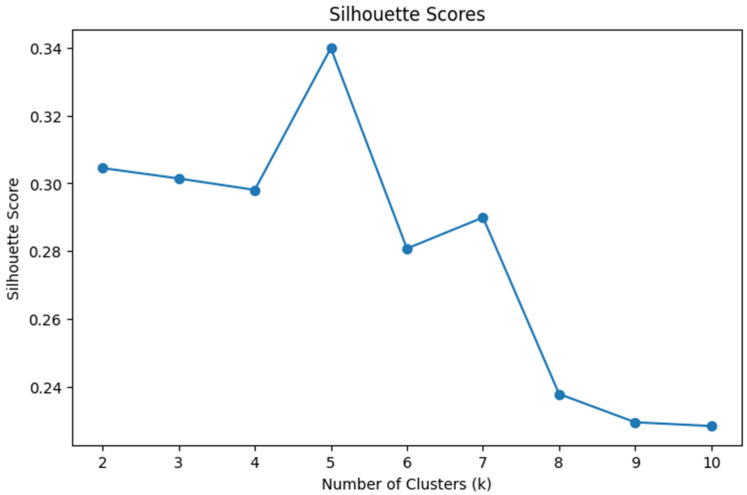
Silhouette Scores.

**Figure 2:**
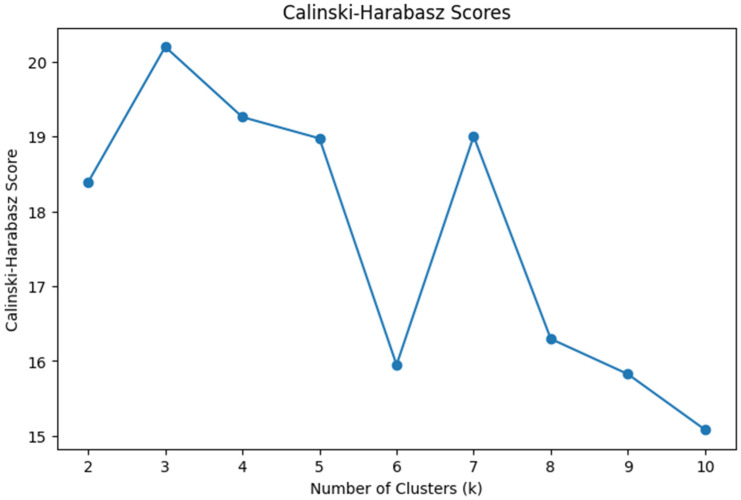
Calinski-Harabasz Scores.

**Figure 3:**
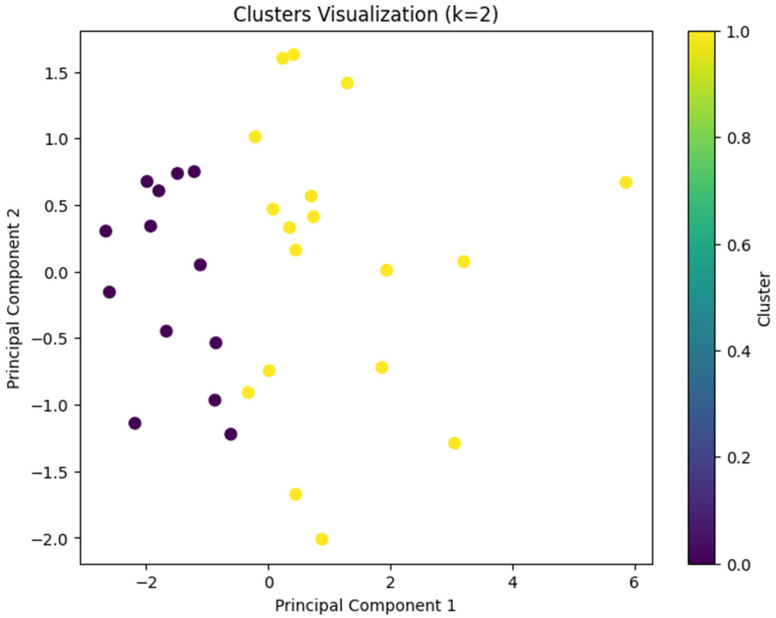
Cluster Visualization.

Cluster analysis was conducted using Python (Version 3.10; Python Software Foundation, 2023) in Google Colab (Google, 2024). We first cleaned the dataset by handling missing values through mean imputation. Numerical variables including interaction with content, instructor, system, and exam scores were standardized to ensure comparability. To obtain the optimal number of clusters, we performed K-means clustering ranging from two to ten clusters. We used Silhouette score and the Calinski-Harabasz index for assessing the model’s performance. The silhouette score indicates how well each data point fits within its cluster, balancing cohesion and separation. The Calinski-Harabasz score evaluates cluster compactness and the degree of separation between them, with higher values indicating better defined clustering structures. Those two serve as internal validation metrics. To explore student engagement behavior patterns, we applied k-means clustering using five key features: interaction with content, instructor, system, total interaction, and course exam scores. The Silhouette score (see Figure 1) peaks at k = 5 and Calinski-Harabasz index (see Figure 2) peaks at k = 3. Both have a reasonably high score when k = 2. Due to the small sample size of the dataset (*n* = 31) and the simplicity and interpretability of the results, we concluded that two clusters would be optimal and allow us to retain the interpretable solution and meaningful group differences. We used principal component analysis (PCA) to visualize the clusters (see Figure 3).

Based on these features, two distinct clusters emerged:

Cluster 1: High-performance group with lower engagement (*n* = 13)
Cluster 2: Low-performance group with higher engagement (*n* = 18).

We used the Shapiro–Wilk test to assess the normality of exam scores and for separate groups. The results indicated no significant departure from the normality for exam scores (W = 0.95, *p* = 0.21), for the high-performance group (W = 0.94, *p* = 0.47), and the low-performance group (W = 0.98, *p* = 0.98). Equality of variances was assessed using the Brown-Forsythe robust test in STATA with all the *p* values higher than 0.14 suggesting no significant difference in variance between groups. The pooled-variance independent sample test was deemed appropriate. Since our hypothesis was directional claiming that the high-performance group has significantly higher scores than the low performance group. The one-tailed *t* test was appropriate. An independent sample *t*-test confirmed a significant difference in exam scores between the two groups (t(29) = 1.87, *p* = 0.04, one-tailed). In addition, A Welch’s t = test produced consistent result (t(28.97) = 1.98, *p* = 0.03, one tail), supporting the robustness of the results. The effect size was medium, Cohen’s d = 0.68, 95% CI [−0.05, 1.41]. The high-performance group had a mean score of 91.59 (SD = 6.67), while the low-performance group had a mean score of 85.77 (SD = 9.68). These findings supported the cluster classification and suggested a noteworthy pattern: higher LMS engagement did not equate to better academic performance.

**Figure 4:**
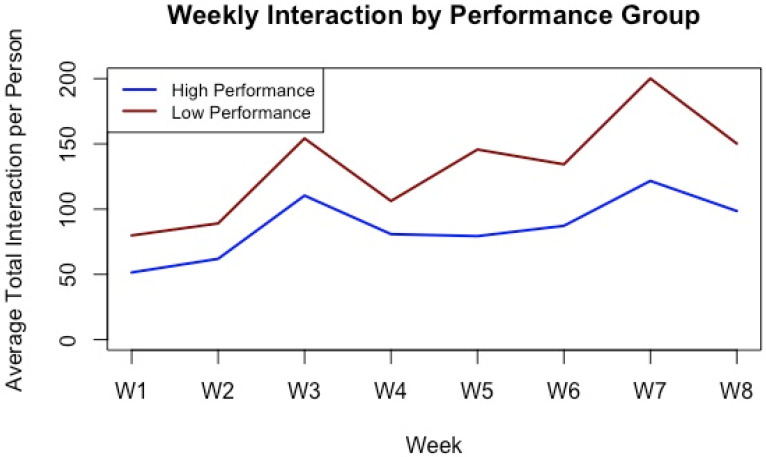
LMS Interaction Trends over Time by Group.

Figure 4 illustrates that both groups showed spikes in LMS activity around key assessments—week 3 (first assignment), week 5 (midterm exam), and week 7 (group project)—indicating that assessments served as primary engagement drivers. However, low-performing students tended to engage more frequently but in a reactive manner, with activity concentrated around deadlines and tapering off afterward. In contrast, high-performing students demonstrated more stable and consistent engagement patterns throughout the semester, suggesting a more proactive and sustained approach to learning.

This finding aligns with prior research indicating that frequent LMS engagement does not always correlate with academic success. For instance, Broadbent (2016) found that students’ self-regulation and time management—rather than high-frequency platform usage—are more predictive of academic achievement. In the current study, the high-performing group exhibited lower interaction frequencies but achieved better academic performance, suggesting that purposeful and efficient engagement may be more beneficial than volume of interaction. Similarly, Bulut et al. (2022) emphasized that performance is best predicted not by total clicks or logins, but by strategic behaviors like timely assignment submissions and quality of engagement. These findings reinforce the importance of distinguishing between quantity and quality of LMS activity in learning analytics research.

### 3.2. Engagement Behavior and Effort Regulation (RQ2)

**Figure 5:**
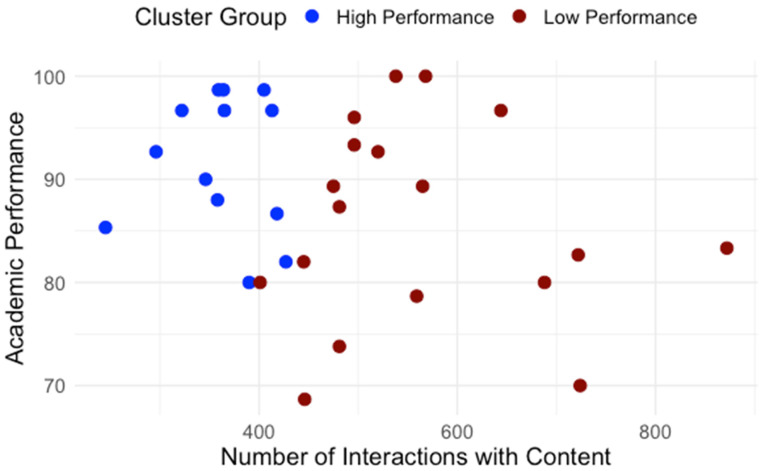
Interactions with Content and Academic Performance.

**Figure 6:**
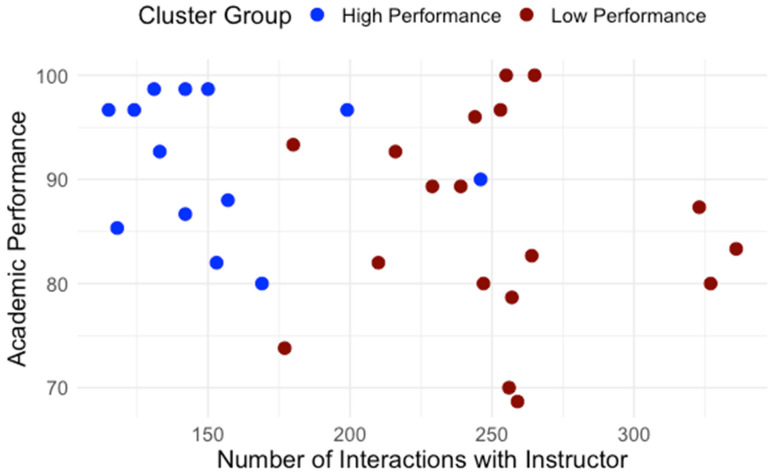
Interactions with Instructor and Academic Performance.

**Figure 7:**
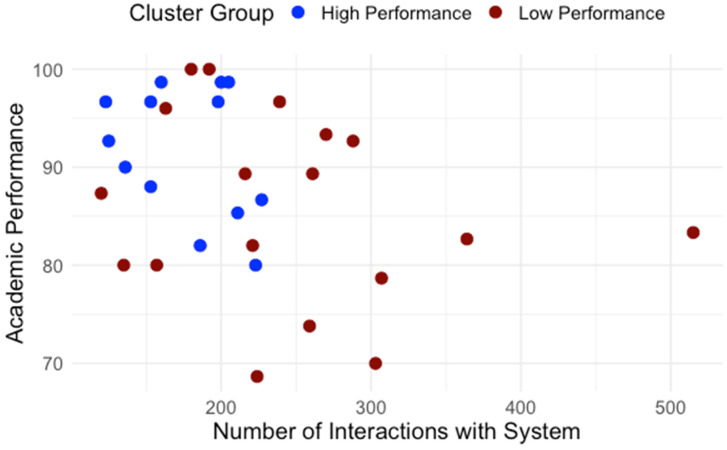
Interactions with System and Academic Performance.

**Figure 8:**
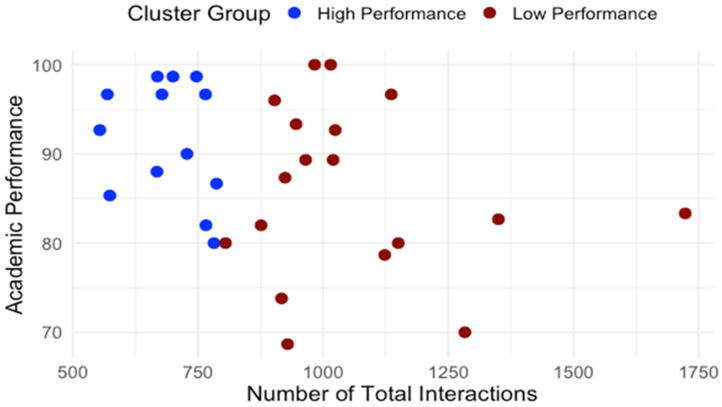
Total Interactions and Academic Performance.

Building on the clusters identified in RQ1, we examined how students differed in engagement patterns and perception of effort regulation. Table 1 details average interaction by type. The normality and homogeneity of variance were assessed for the assumption check. Independent-samples t-tests were conducted to compare learning interaction patterns between high- and low-performance groups. As shown in Table 1, high and low performance groups showed significant differences regarding interaction with content, system, instructor and total interaction. To control multiple comparisons across the four interaction measures, a Bonferroni correction was applied (a = 0.0125). After the adjustment, group differences in interaction with content, instructor, and total interaction remained significant while the groups difference in interaction with the system did not. Across all categories, low-performing students interacted significantly more, especially with content and instructor features. Figure 5 presents the distribution of interactions with content for high- and low-performance groups. Figure 6 presents the distribution of interactions with instructor interactions for high- and low-performance groups. Figure 7 presents the distribution of interactions with system for high- and low-performance groups. Figure 8 presents the distribution of total interactions for high- and low-performance groups. Yet, as Figure 5, Figure 6, Figure 7 and Figure 8 visually demonstrate, more frequent interaction did not translate to stronger performance.

**Figure 9:**
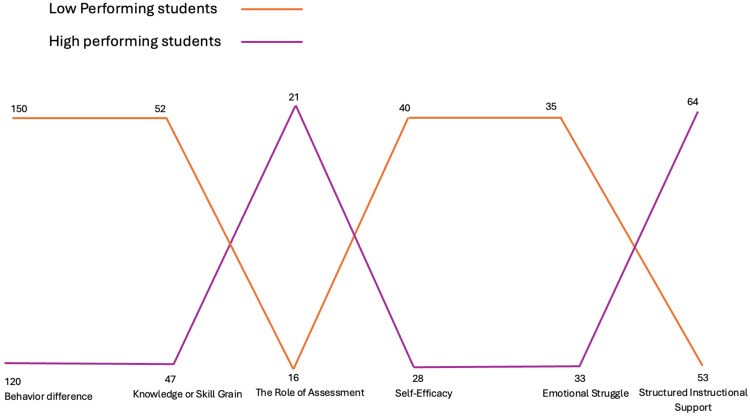
MAXQDA Portfolio View—Theme Distribution by Group.

We further contextualized these differences using interview data. Six major themes emerged from the qualitative analysis (Figure 9): behavioral differences in engagement, the role of assessment, emotional struggle, self-efficacy, knowledge or skill gain, and structured instructional support. Table 2 provides brief definitions and representative quotes for each theme.

#### 3.2.1. Behavioral Differences in Engagement

We identified the theme of Behavioral Differences in Engagement to provide direct insight into the behavioral engagement patterns seen in the quantitative data. Many low-performing students described their LMS engagement as a response to immediate demands—such as upcoming deadlines or confusion—rather than a sustained, strategic routine. One student noted, “I accessed it every time we had the lecture, and every time we had assignments and group work… I downloaded them and accessed them on One Drive” [0403L_addition]. This pattern was echoed by others who used LMS features primarily to catch up or clarify content. “I mostly watched the class recordings… that was my go-to because sometimes I missed things in class. I’d go back and take screenshots” [1201L_post]. “I didn’t really organize my time well. I just did stuff when it was due, not before” [1902L_post]. This quote illustrates a form of passive, catch-up-based engagement, where Canvas is used heavily but mostly to recover or keep up.

In contrast, high-performing students shared more proactive habits, describing LMS interaction as part of a consistent, self-directed workflow, “I would do like 30 min each day before, leading up so that it wasn’t all the night before or the day of…” [0603H_addition]. Their responses reflected a deliberate effort to maintain steady progress. “I maximize my classroom time, because I don’t have a lot of time outside of class… I show up… focused and ready to engage” [0503H_addition]. “Usually, I try to get ahead of deadlines, like completing things a day early. That way I can double check everything before submission” [1702H_post]. These students used the LMS features as part of a structured learning strategy, not just to meet deadlines but to maintain regular progress. These accounts support the quantitative finding that fewer interactions do not necessarily mean lower quality engagement—in fact, high-performers seemed to engage more purposefully and effectively.

#### 3.2.2. The Role of Assessment

A second theme across both groups was the influence of assessments which played a crucial role in shaping student engagement behaviors. Both groups increased LMS activity around exams and assignments, but low-performing students often relied on assessments as motivators or checkpoints for understanding. One low-performing student shared, “Assignments were very helpful… that helped balance your grade out throughout the semester” [1902L_post]. Another noted, “It gave me opportunity to even ask questions, and when I made mistakes, she was able to give feedback” [1602L_post]. Similarly, assessments also served as opportunities to clarify confusion and receive targeted help. “Before the test, I would go back to every assignment and try to understand what I missed, and sometimes I’d email the instructor to confirm.” [1201L_post].

High-performing students, in contrast, used assessments more for self-checking. One explained, “The midterm… was helpful to make sure I knew some of the competencies” [1702H_post]. And another emphasized how assignments supported independent learning. “Assignments are first. Getting that independent practice in—that was helpful” [0503H_addition]. “I would review my mistakes from the last quiz or test just to track what I’m doing wrong before the next one.” [0603H_addition].

As we can see, both groups increased LMS engagement around assignments and exams, but for different reasons. Low-performing students used assessments as external motivators or sought feedback to recover from mistakes. High performers viewed assessments as checkpoints to self-evaluate progress. This is confirmed by Temiyasathit et al. (2016) as these researchers observed that assessment-driven engagement varies in quality and intent.

#### 3.2.3. Emotional Struggle

Emotional challenges were present for both low- and high-performing students, although the sources and experiences varied. This theme contextualized why some students, especially in the low-performing group, may engage more but still struggle academically. For many low-performing students, the course triggered early anxiety—especially when confronting unfamiliar statistical terms or software tools. One student mentioned, “My biggest challenge was just understanding the terminology… I’ve had to buy, like, a statistics glossary” [1203L_addition]. This anxiety was further heightened by the pressure to grasp concepts quickly, which led some to seek external aid. “At the beginning, I cried after the first class because I didn’t know what an R file was or how to use it” [1902L_pre]. “I would open the assignments and just freeze sometimes. It looked so different from anything I’ve done before.” [0401L_pre].

High-performing students also reported experiencing stress, but their concerns were often tied to time management and balancing academic responsibilities. One student reflected, “My only challenge is time—really having that time outside of class to review and practice” [0503H_addition]. This time pressure often came from their effort to maintain steady performance while juggling other commitments. “Sometimes I’d plan to practice but just couldn’t find the time, and that frustrated me” [0603H_post]. “Managing this with research and my assistantship was a lot. I had to be very intentional with planning” [1702H_post]. For both groups, however, the instructor’s teaching style, supportive feedback, and structure of the course helped ease these emotional struggles over time.

These findings demonstrate that while low-performing students engaged more frequently with the LMS, this behavior did not translate into higher academic achievement. These students exhibited more reactive patterns of interaction, especially around assessments, suggesting a reliance on external cues and structured support to maintain engagement. In contrast, high-performing students interacted less often but more consistently and intentionally, supporting previous literature on self-regulated learning (Broadbent, 2016). The qualitative data reinforced this distinction, revealing that low-performing students often used Canvas features like reminders and recordings to catch up or clarify confusion, whereas high-performers integrated LMS usage into a broader system of proactive time management and preparation. These results align with Temiyasathit et al. (2016), who observed that LMS engagement often peaks around assessments but varies significantly in quality depending on students’ learning approaches. Together, these insights underscore the importance of interpreting LMS interaction not only by volume but by context and strategy.

### 3.3. Perceived Learning Outcomes and Instructional Impact (RQ3)

For our third research question, we examined how students interpreted their learning experience, particularly regarding self-efficacy, PBL impact, and perceived outcomes. We did an assumption check including the normality and homogeneity of variance. For those who met the assumption checks (i.e., social presence and self-efficacy pre), we conducted independent sample t tests and reported the Cohen’s d CI. For those that did not meet the normal distribution (i.e., social presence, teaching presence, impact of PBL, motivation, self-efficacy post), we ran a Mann–Whitney U test as the nonparametric alternatives and probability-based effect size CIs (like Harrell’s c) were reported. Table 3 demonstrated that no statistical significance was detected among high and low performance groups among all variables including self-efficacy and impact of PBL. Interestingly, we observed the pattern that low-performing students rated the impact of PBL higher (M = 20.96 vs. 18.89 for high performers) and showed greater gains in self-efficacy from pre- to post- survey (M diff = 18.8 vs. 13.04 for high performers). Although not statistically significant, these results suggest a potential trend toward deeper affective and cognitive shifts in the lower-performing group.

**Figure 10:**
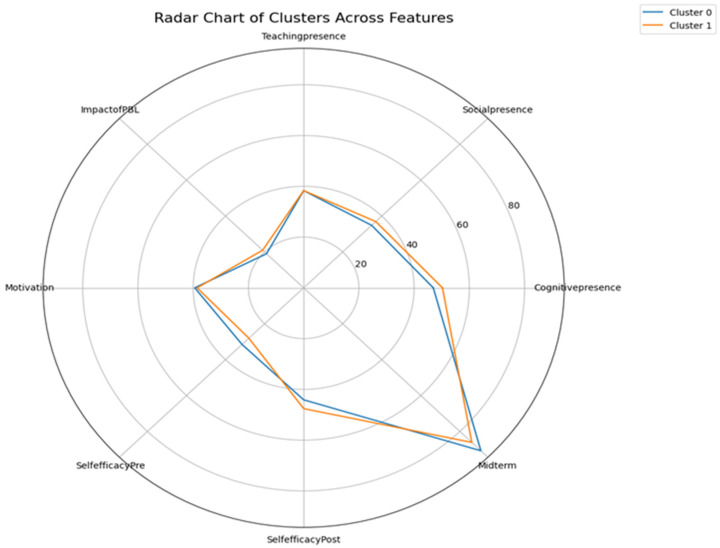
Radar Chart of Cluster across Features.

Figure 10 complements these findings by visualizing average post-survey scores across key domains. The radar chart shows that while both groups reported comparable outcomes in motivation, teaching presence, and cognitive presence, low-performing students had slightly elevated perceptions of social presence and PBL impact. This visualization reinforces the pattern that although academic performance differed, students with initially lower scores experienced notable gains in affective and cognitive domains.

To further interpret these quantitative trends, qualitative interview data provided important context. Students’ reflections aligned with and expanded upon the survey results, revealing three key themes: knowledge and skill gain, self-efficacy, and structured instructional support. These themes help clarify the deeper, non-statistical benefits reported by students with lower initial performance and provide insight into how PBL and course structure supported learning across both groups.

#### 3.3.1. Knowledge or Skill Gain

Students across performance groups expressed that the course deepened their understanding of core statistical principles and practical applications. Many low-performing students acknowledged gaps in foundational knowledge at the outset but credited the course structure with helping them develop stronger conceptual and procedural skills. “I didn’t really get like deep understanding of what we were being taught… but now it’s helping me to really understand. This is more meaningful” (0401L_pre). This shift from surface-level understanding to meaningful comprehension was echoed by others. “When I started, I didn’t know anything about R… now I can run t-tests and even explain them” [1902L_post]. “The practice in class made a big difference for me. It wasn’t just theory—we actually did it” [0403L_post]. This theme was consistent with the purpose of the PBL model, which emphasized applied problem-solving. Students appreciated real-world contexts and exposure to statistical software like SPSS (Version 29.0.2.0; IBM Corp., 2025) and R, which helped bridge theory and practice. High-performing students also reflected positively on the course’s role in advancing their technical and analytical skills. “It challenged me to not just use formulas but understand why I was using them” [0503H_addition]. “This is the first stats course where I’ve felt confident doing actual data analysis on my own” [0603H_post]. These comments support the finding that the course was effective in promoting both procedural fluency and conceptual mastery, regardless of prior experience.

#### 3.3.2. Self-Efficacy

Although statistical differences were not detected, quantitative results showed that low-performing students demonstrated greater increases in self-efficacy from pre- to post, evidencing by mean difference. This trend was echoed in qualitative accounts. Low-performing students in particular reported gaining confidence through structured materials and instructor support. “With what we’ve learned… I would say I’m at a strongly confident level. Maybe on a scale of one to five, I would say I’m at a four” [0401L_pre]. This upward shift in confidence was a common experience among several low performers. “I never thought I could do this, but now I feel like I can handle stats in my research” [1602L_post]. “I’ve gone from being scared of numbers to actually liking R coding” [0403L_post]. These comments suggest that while high-performing students entered the course with existing confidence, it was the low-performing students who experienced more transformational growth.

High performers, meanwhile, reflected a sense of sustained or reinforced self-efficacy rather than dramatic improvement. “I came in feeling pretty confident, but the structure kept me focused and consistent” [1702H_post]. “It confirmed I’m on the right track, especially when I did well on the midterm” [1702H_post]. These insights reinforce that while high-performers relied on prior competence, low-performers benefited greatly from the instructional design in building their self-belief.

Our findings align with previous studies emphasizing that PBL environments can be especially beneficial for students with lower prior confidence or skill, as they provide contextualized learning and scaffolded support (Bihari et al., 2021; Bukumiric et al., 2022). The increase in self-efficacy among lower-performing students also supports Bandura’s (1997) assertion that mastery experiences are one of the most powerful sources of efficacy beliefs.

#### 3.3.3. Structured Instructional Support

We found that both groups valued the layered instructional design, but it was especially meaningful for those who needed extra reinforcement. Students mentioned the value of lecture notes, class practice, and supplementary videos in helping them grasp challenging concepts. “The structure… professor would share the notes, then go over them, and then we would have class practices, maybe a video to support that… The entire approach is beneficial” [0401L_pre]. This appreciation for structure and repetition was echoed across the comments of several low-performing students. “She repeated the concepts in different ways. That helped me because I learn slowly” [1203L_post]. “Reminders and Canvas notifications really kept me on track” [1902L_post].

Even when external resources like YouTube were used, students appreciated the predictability and clarity of the course structure. The blend of synchronous and asynchronous support helped build a reliable learning environment. High-performing students also noted that the organization and pacing of the course supported their learning, though their engagement was often more self-directed. “Having everything in one module helped me move ahead at my own pace” [0503H_addition]. “The coding walkthroughs were short and straight to the point—that’s what made them effective” [1702H_post].

Participant responses underscore that while structured support benefits all learners, it is especially critical for students who need clearer scaffolding and reinforcement throughout the semester. Structured instructional components such as live coding, group discussions, and repeated practice helped students build both confidence and competence, echoing Varadarajan and Ladage’s (2022) findings that guided instruction enhances students’ perceived control over complex learning tasks. Overall, our qualitative and quantitative results together suggest that the PBL model, when paired with consistent structure, can help close the confidence and skill gap between lower- and higher-performing students.

## 4. Conclusions, Limitations, and Future Directions

This study examined how students with different performance levels engaged in a graduate statistics course delivered through PBL and an LMS (Canvas). Drawing on LMS log data and student interviews, the analysis revealed a paradox: low-performing students showed higher levels of online activity, often rewatching recordings, revisiting assignments, and reviewing materials to keep pace. High-performing students, in contrast, accessed the LMS less frequently but used it more deliberately, preparing ahead of time and focusing their effort during class. These results caution against the assumption that more LMS activity equates to stronger engagement. Instead, frequent use may indicate difficulty rather than mastery. Engagement patterns after assessments reinforced this distinction, as low-performing students’ activity declined sharply while high-performing students maintained consistent involvement.

Despite their differences, both groups valued structured instructional support, especially live coding, clear materials, and timely feedback. These elements not only supported learning but also helped reduce anxiety and improve self-efficacy, particularly for those with lower initial confidence. Ultimately, our findings underscore the importance of designing courses that accommodate diverse learning needs. A well-organized LMS and scaffolded PBL instruction can empower all students, especially those who might otherwise fall behind—to succeed with confidence.

This study has several limitations. The sample was small and drawn from a single graduate-level statistics course, which limits generalizability. The setting was also specific to one institution and discipline, so engagement patterns may not extend to other contexts. In addition, only 19 of the 31 students agreed to be interviewed. The absence of perspectives from the 12 students who declined may have introduced bias if their experiences differed systematically from those who participated. Another limitation relates to the use of LMS log data: student activity records may include random clicks or off-task behaviors such as mind wandering, which are still recorded as time on the platform. Finally, while combining LMS log data with student interviews provided valuable insights, the study may not account for all factors influencing student learning and motivation.

Future studies should examine a larger and more diverse set of courses to test whether the paradox observed here holds across different disciplines and institutions. It would also be useful to compare how alternative instructional designs influence the link between LMS engagement and learning outcomes. Beyond the classroom, learning analytics could be applied in hybrid learning environments to evaluate extension and outreach programs, which are widely used in U.S. agriculture. Such work could provide practical evidence of how technology can support more flexible and effective learning in real-world contexts.

## Figures and Tables

**Table 1:** Learning interaction pattern among high vs. low performance groups (n = 31).

	Interaction with Content	Interaction with Instructor	Interaction with System	Total Interaction
High performance (*n* = 13)	362.15 (14.44)	152.23 (10.02)	176.92 (10.24)	691.31 (22.89)
Low performance (*n* = 18)	562.28 (28.28)	252.06 (10.35)	245.58 (22.02)	1059.56 (51.25)
Cohen’s d	−8.5	−9.773	−3.79	−8.79
95% CI	[−10.79, −6.19]	[−12.39, −7.16]	[−5.00, −2.58]	[−11.16, −6.4]
*p* value of *t* test	<0.001 ***	<0.001 ***	0.019 *	<0.001 ***

Note * *p* < 0.05, *** *p* < 0.001.

**Table 2:** Summary of the Six Emergent Themes, Grouped by Research Question.

RQ	Theme	Brief Definition	Representative Quote
RQ2—Engagement Patterns	Behavioral Differences in Engagement	Differences in students’ learning habits and LMS usage between high- and low-performers.	“I didn’t really organize my time well. I just did stuff when it was due, not before.” [1902L_post]
	The Role of Assessment	How evaluations shaped motivation and engagement behaviors.	“Before the test, I would go back to every assignment and try to understand what I missed.” [1201L_post]
	Emotional Struggle	Students’ stress, anxiety, and coping strategies during the course.	“At the beginning, I cried after the first class because I didn’t know what an R file was.” [1902L_pre]
RQ3—Perceived Learning Outcomes	Knowledge or Skill Gain	Perceived growth in understanding of statistics.	“This is the first stats course where I’ve felt confident doing actual data analysis on my own” [0603H_post].
	Self-Efficacy	Students’ confidence in their ability to learn and complete tasks.	“I came in feeling pretty confident, but the structure kept me focused and consistent” [1702H_post].
	Structured Instructional Support	The perceived need for guidance, pacing, or reminders to stay on track.	“The structure… professor would share the notes, then go over them, and then we would have class practices, maybe a video to support that… The entire approach is beneficial” [0401L_pre].

**Table 3:** Comparison of average post-survey ratings by group.

	Cognitive Presence	Social Presence	Teaching Presence	Impact of PBL	Motivation	Self-Efficacy Pre	Self-Efficacy Post
High performance (*n* = 13)	46.95 (8.60)	34.53 (5.63)	38.24 (1.90)	18.89 (6.59	39.12 (7.24)	30.81 (11.39)	43.85 (11.83)
Low performance (*n* = 18)	50.08 (8.72)	37.02 (7.02)	38.35 (7.67)	20.96 (3.17)	38.85 (7.79)	28.77 (12.33)	47.57 (7.78)
Mann–Whitney U test	0.166		0.73	0.57	0.82		0.59
*p* value of *t* test		0.30				0.64	
Cohen’s d 95% CI		[−1.104, 0.336]				[−0.544, 0.885]	
Harrell’s c 95% CI	[0.47, 0.68]		[0.41, 0.63]	[0.42, 0.64]	[0.40, 0.62]		[0.41, 0.65]

## Data Availability

The original data presented in the study are openly available in Texas Data Repository at https://doi.org/10.18738/T8/PMXCO6 (accessed on 19 September 2025).
